# P319: Interest and feasibility of weekly monitoring of blood transfusion activities in Benin

**DOI:** 10.1186/2047-2994-2-S1-P319

**Published:** 2013-06-20

**Authors:** L Anani, M Atinkpinda, D Aidéwou, G Magnidet, E Padonou, O Ahignissè, G Ahissou, U Djodjo, R Laguide, MR Durand, F Ahlonsou, E Lozes, M Godjo

**Affiliations:** 1 National Agency for Blood Transfusion , Cotonou, Benin

## Introduction

The National Agency for Blood Transfusion (**NABT**) was created to ensure the smooth functioning of the transfusion system and made available in real time, quality blood products.

## Objectives

In order to have a dashboard for correct decisions in real time, the ANTS team leader has initiated a weekly collection of data from the Blood Transfusion Departmental Centers (BTDC). This abstract discusses the implementation of this activity and its benefits, challenges and perspectives.

## Methods

Three operational indicators for an objective assessment of activities and their impact have been identified as following: the number of validated pockets in the week; the overall satisfaction of the blood products requests; the proportion of the blood pockets wasted after biological qualification.

Data, mostly from BTDC are sent electronically to the NABT General Office every Friday. They concern the activities from one thursday to the next one. The data are analyzed by BTDC from week to week as their synthesis at central level.

## Results

46,373 units of blood were collected by all the BTDC from march to december 2012. We have, after post qualification rejection of 13.21% of collected pockets, satisfied 86.64% requests for blood products.

We were able to have indicative data for transfusion activities in the country and follow their weekly evolution. "Dangerous" sampling sites at departmental level have been identified with initiation of communication activities for behavior change. Inter-departmental transfusion activities comparison with the possibility of technical orientation or financial support has been possible.

The current difficulties are irregular and unstable internet connection. Our perspectives are: the sustainability of the weekly monitoring and improving serological markers monitoring.

## Conclusion

Weekly monitoring: allows transfusion activities control and transform transfusion activities to a blood transmitted diseases observatory. Thus the quality blood products supply is not only a guarantee for the safety of patients, but also contributes to the fight against blood transmitted diseases.

## Disclosure of interest

None declared

